# Treatment with Imatinib in NSCLC is associated with decrease of phosphorylated PDGFR-*β* and VEGF expression, decrease in interstitial fluid pressure and improvement of oxygenation

**DOI:** 10.1038/sj.bjc.6603366

**Published:** 2006-09-26

**Authors:** G Vlahovic, Z N Rabbani, J E Herndon, M W Dewhirst, Z Vujaskovic

**Affiliations:** 1 Duke University Medical Center, P.O. Box 3335, Durham, NC 27710, USA

**Keywords:** lung cancer, interstitial fluid pressure, hypoxia, imatinib, VEGF

## Abstract

Elevated intratumoral interstitial fluid pressure (IFP) and tumour hypoxia are independent predictive factors for poor survival and poor treatment response in cancer patients. However, the relationship between IFP and tumour hypoxia has not yet been clearly established. Preclinical studies have shown that lowering IFP improves treatment response to cytotoxic therapy. Interstitial fluid pressure can be reduced by inhibition of phosphorylated platelet-derived growth factor receptor-*β* (p-PDGFR-*β*), a tyrosine kinase receptor frequently overexpressed in cancer stroma, and/or by inhibition of VEGF, a growth factor commonly overexpressed in tumours overexpressing p-PDGFR-*β*. We hypothesised that Imatinib, a specific PDGFR-*β* inhibitor will, in addition to p-PDGFR-*β* inhibition, downregulate VEGF, decrease IFP and improve tumour oxygenation. A549 human lung adenocarcinoma xenografts overexpressing PDGFR-*β* were grown in nude mice. Tumour-bearing animals were randomised to control and treatment groups (Imatinib 50 mg kg^−1^ via gavage for 4 days). Interstitial fluid pressure was measured in both groups before and after treatment. EF5, a hypoxia marker, was administered 3 h before being killed. Tumours were sectioned and stained for p-PDGFR-*β*, VEGF and EF5 binding. Stained sections were viewed with a fluorescence microscope and image analysis was performed. Imatinib treatment resulted in significant reduction of p-PDGFR-*β*, VEGF and IFP. Tumour oxygenation was also significantly improved. This study shows that p-PDGFR-*β*-overexpressing tumours can be effectively treated with Imatinib to decrease tumour IFP. Importantly, this is the first study demonstrating that Imatinib treatment improves tumour oxygenation and downregulates tumour VEGF expression.

Non-small-cell lung cancer (NSCLC) accounts for approximately 80% of all lung cancers and is the leading cause of cancer-related deaths worldwide (Jemal *et al*, 2005). The response rate for first-line therapy in NSCLC is 19% with 1- and 2-year survival rates in the range of 30–36% and 10–16%, respectively (Schiller *et al*, 2002). Factors thought to contribute to poor tumour response to chemotherapy and/or radiation therapy are limitations in drug delivery resulting from elevated intratumoral interstitial fluid pressure (IFP) (Stohrer *et al*, 2000) and reduced treatment sensitivity due to tumour hypoxia (Brizel *et al*, 1997).

One of the physiological challenges in drug delivery to tumour cells is its heterogeneous blood supply and abnormal microvascular permeability that leads to increased IFP and poor drug penetration. Elevated IFP is a hallmark of solid tumours and has been identified as an independent predictor for poor radiation treatment response in cervical cancer (Netti *et al*, 1996; Griffon-Etienne *et al*, 1999; Milosevic *et al*, 2004).

In addition to IFP, tumour hypoxia has been shown to be an independent predictor for local control and patient survival following radiation therapy for carcinoma of the cervix and head and neck cancer (Brizel *et al*, 1999; Birner *et al*, 2000; Koukourakis *et al*, 2001; Bachtiary *et al*, 2003; Nordsmark and Overgaard, 2004). Comparison of IFP and *p*O_2_ measurements in patients with cervical cancer demonstrate that IFP is a stronger predictor than hypoxia for pelvic recurrence and disease-free survival (Milosevic *et al*, 2004). Although the relationship between IFP and *p*O_2_ is not fully understood, hypoxia is believed to be associated with an increased IFP in solid tumours (Taghian *et al*, 2005).

Phosphorylated PDGFR-*β*, a stromal tyrosine kinase receptor, is recognised as a strategic target for lowering IFP in tumours overexpressing PDGFR-*β*. PDGFR-*β* is overexpressed in 50–80% of patients with NSCLC (Kawai *et al*, 1997). When phosphorylated, PDGFR-*β* contributes to increased IFP by enhanced stimulation of stromal growth, which disrupts interstitial homeostasis (Heuchel *et al*, 1999; Ostman, 2004) and by participation in tumour angiogenesis (Pietras *et al*, 2001; Labrecque *et al*, 2005). Although VEGF is the predominate angiogenic factor that promotes endothelial cell (EC) proliferation, migration, vessel sprouting, vessel remodelling and vascular permeability (Dvorak *et al*, 1995), p-PDGFR-*β* is required for pericyte and smooth muscle cell recruitment and for the subsequent completion of the angiogenic process (Nykanen *et al*, 2005). Preclinical data have reported that IFP can be reduced by p-PDGFR-*β* inhibition using Imatinib (STI571, Gleevec®, Novartis Pharmaceuticals) (Pietras *et al*, 2001). Imatinib is a PDGFR-*β*-specific small-molecule tyrosine kinase inhibitor. We hypothesised that Imatinib, as a consequence of p-PDGFR-*β* inhibition, will affect angiogenesis, decrease IFP and improve tumour oxygenation in an NSCLC xenograft.

## MATERIALS AND METHODS

### Animal NSCLC model

Adult female nude mice were used as host animals for xenograft tumours. Tumours were grown from exponentially growing A549 cell cultures. The A549 cell line was derived from human lung carcinoma and was purchased from ATCC. Cells were grown in RPMI 1640 media supplemented with 5% bovine calf serum. Cells (∼5.0 × 10^5^) suspended in 200 *μ*l of HBSS were injected subcutaneously into the right hind limb of each mouse.

Tumour growth was monitored twice weekly until they reached an average volume of 100 mm^3^. Tumour volume (*V*) was determined by the following equation: *V*=(*L* × *W*^2^) × 0.5, where *L* is the length and *W* is the width of the tumour. Tumour-bearing animals were then randomised into control (saline) and treatment groups. There were 10 animals in each treatment group. Imatinib (STI571, Gleevec®), 50 mg kg^−1^, was administered to tumour-bearing mice once a day for 4 days. The study was repeated twice. Imatinib was commercially obtained in a 100 mg tablet form, crushed and re-suspended in saline and given via gavage.

Animal experiments were approved by the Institutional Committee on Research Animal Care and were performed in strict accordance to the Interdisciplinary Principles and Guidelines for the Use of Animals in Research, Marketing and Education (New York Academy of Sciences, New York, NY, USA) and in accordance with the UKCCCR guidelines (Anon, 1988).

### IFP measurements

Animals were anaesthetised using nembutal (75 mg kg^−1^). Interstitial fluid pressure measurements were performed for each animal before and 3 h after the last saline or Imatinib treatment. Tumour IFP was measured using the wick-in-needle technique as described previously (Boucher *et al*, 1991). A standard 23-gauge needle filled with nylon floss and saline, supplemented with 50 IE ml^−1^ of heparin, was inserted into the centre of the tumour and connected to a pressure transducer (World Precision Instruments Inc., Sarasota, Florida, USA). This set-up enabled continuous and stable recording of fluid pressure. Fluid communication between the needle and the transducer was confirmed by compression and decompression of the tubing during each measurement. Tumour IFP was determined by calculating the mean of two readings. Interstitial fluid pressure values in muscle and subcutaneous tissue served as controls.

### Hypoxia marker EF5

Mice received an intravenous (i.v.) injection (0.2 ml of 10 mM EF5) of the hypoxia marker EF5 (2-(2-nitro-1*H*-imidazole-1-yl)-*N*-(2,2,3,3,3-pentafluoropropyl) acetamide), generously provided by Dr Cameron Koch, University of Pennsylvania, 3 h before tumour excision. After killing the animals, the tumour specimens were removed and snap frozen in liquid nitrogen. Specimens were stored at −80°C until sectioned.

### Immunohistochemical staining

Frozen specimens were cut into 10–12 *μ*m sections using a LEICA CM 1850 cryotome (Meyer Instruments Inc., Houston, TX, USA). Serial tissue sections were placed onto poly-L-lysine-coated slides (Polysciences Inc., Warrington, PA, USA). Consecutive tissue sections were cut at the largest circumference of the tumour. The sections were fixed in 4% paraformaldehyde in PBS (pH 7.2) for 1 h and incubated in the presence of primary antibodies. Primary antibody signals were visualised and enhanced by fluorescence-conjugated secondary antibodies to obtain sufficient fluorescence signal for imaging. Sections were rinsed three times for 2 min in PBS between each consecutive step of the staining procedure. Unless stated otherwise, all antibodies were diluted in PBS with 0.1% bovine serum albumin and 0.1% Tween-20 (PBS-BT). Omission of the primary antibody served as a negative control.

#### p-PDGFR-*β*

Tissue sections were incubated overnight at 4°C with goat polyclonal antibody to p-PDGFR-*β* (Santa Cruz Biotechnology, Santa Cruz, CA, USA) diluted 1 : 400. The sections were then incubated for 1 h at room temperature with Texas Red-conjugated donkey anti-goat 1 : 200 (Jackson Immunoresearch, West Grove, PA, USA).

#### EF5

Tumour hypoxia was assessed immunohistochemically. EF5, a 2-nitroimidazole, is selectively retained in hypoxic tissues because it is metabolised to a free radical form in cells that binds to proteins to form specific residues in tissue that can be recognised by antibodies (Yuan *et al*, 2006). EF5 binding was visualised using a fluorochrome (Cy3) conjugated to the ELK3-51 antibody. The sections were incubated for 1 h, with mouse monoclonal antibody diluted 10 *μ*l ml^−1^ (Jackson Immunoresearch, PA, USA).

#### Endothelial cell marker (CD-31)

Tissue sections were incubated overnight at 4°C with rat anti-mouse CD31 antibody (BD Biosciences, San Jose, CA, USA) diluted 1 : 200 and then incubated for 1 h at room temperature with FITC-conjugated goat anti-rat antibody 1 : 100.

### VEGF

Endogenous peroxidase activity was quenched with 3% hydrogen peroxide for 15 min. Tumour sections were then blocked with 10% donkey serum for an additional 15 min. Sections were incubated overnight at 4°C with rabbit polyclonal antibody against VEGF diluted 1 : 100 (Santa Cruz Biotechnology, Santa Cruz, CA, USA). After rinsing 3 × 5 min with PBS, biotinylated donkey anti-rabbit antibody (Jackson Immunoresearch, PA, USA) was applied for 30 min at room temperature. The slides were washed with PBS for 3 × 5 min, followed by application of an avidin–biotin complex (Vectastain ABC kit, Vector Lab Inc., Burlingame, CA, USA). Location of the reaction was visualised with 3, 3′-diaminobenzidine tetrahydrochloride (DAB chromogen, Vector Lab Inc., Burlingame, CA, USA).

### Image analysis

The tumour sections were quantitatively analysed with a semiautomatic method based on a computerised digital image analysis system. A high-resolution intensified solid-state camera on a fluorescence microscope (Axioskop Zplus, Carl Zeiss Inc., Germany) with a computer-controlled motorised stepping stage was used. Each tumour cross-section was sequentially scanned two times at × 50 magnification, using different filters for the FITC (green) and Texas Red and Cy3 (red) signals.

After each scan, a high-resolution composite digital image was reconstructed from the individual microscopic fields. The whole scanning procedure thus yielded two composite images from each tumour section. The thresholds for positive staining were set to be background staining for each individual marker.

Haematoxylin and eosin staining on previously immunostained sections permitted contour lines to be drawn around the tumour area and exclusion of necrotic areas that were excluded from the quantitative analysis of each marker. The positively stained regions (EF5 or p-PDGFR-*β*) were segmented using a single threshold value for all slides and all areas >2 pixels (72 *μ*m^2^) in size were considered as positive regions. The stained fraction (%) for each marker was then calculated by dividing the stained area by the total area to give the hypoxic fraction and the relative p-PDGFR-*β* expression. To assess mean vessel density (MVD), the number of vessels, determined by positive CD31 staining, were counted in 6–8 fields and averaged to get a mean value for each tumour. The representative images for publication have been adjusted for brightness, contrast and colour balance for ease of viewing, but quantification was performed without any manipulation of the above-mentioned parameters.

### Statistical analysis

The paired-*t*-test was applied to compare pre- and post-treatment IFP measurements between groups. A two-sample *t*-test was used to compare saline and Imatinib groups relative to the following outcome measures: PDGFR-*β*, EF5, EGF and MVD. Pearson's correlation, or simple linear regression, was used to examine the relationship between outcome measures within each treatment arm. Data are presented as mean±s.e.m. Significant differences between groups were determined with a Student *t*-test. In all cases, *P*⩽0.05 was considered statistically significant.

## RESULTS

**Figure 1:**
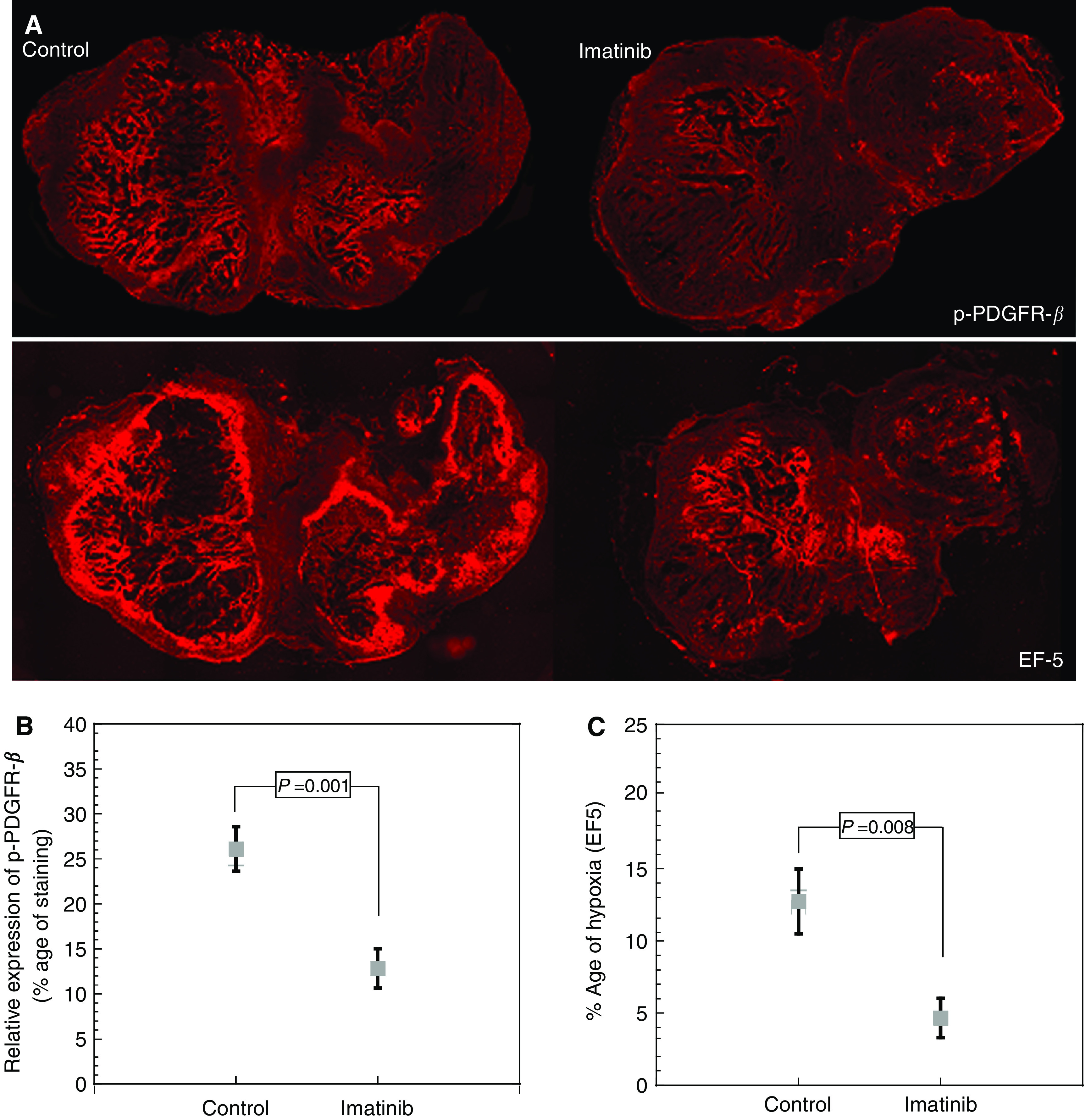
(**A**) Photomicrographs of p-PDGFR-*β* expression and tumour hypoxia (EF5) in control and after treatment with Imatinib. (**B**) p-PDGFR-*β* expression in tumour after 4 consecutive days of treatment with Imatinib (50 mg kg^−1^) (mean±s.e.m.). (**C**) Tumour hypoxia after 4 days of treatment with Imatinib (50 mg kg^−1^) (mean±s.e.m.)

Before the initiation of experiments, it was determined that the A549 xenograft overexpressed p-PDGFR-*β* (Figure 1A). After 4 days of treatment, the percentage of expression of p-PDGFR-*β* in tumour sections was 26±2.5% (mean±s.e.m.) for control samples and 13±2.2% (mean±s.e.m.) for the Imatinib-treated group. This decrease in p-PDGFR-*β* expression after Imatinib treatment was statistically significant (*P*=0.001) (Figure 1B).

**Figure 2:**
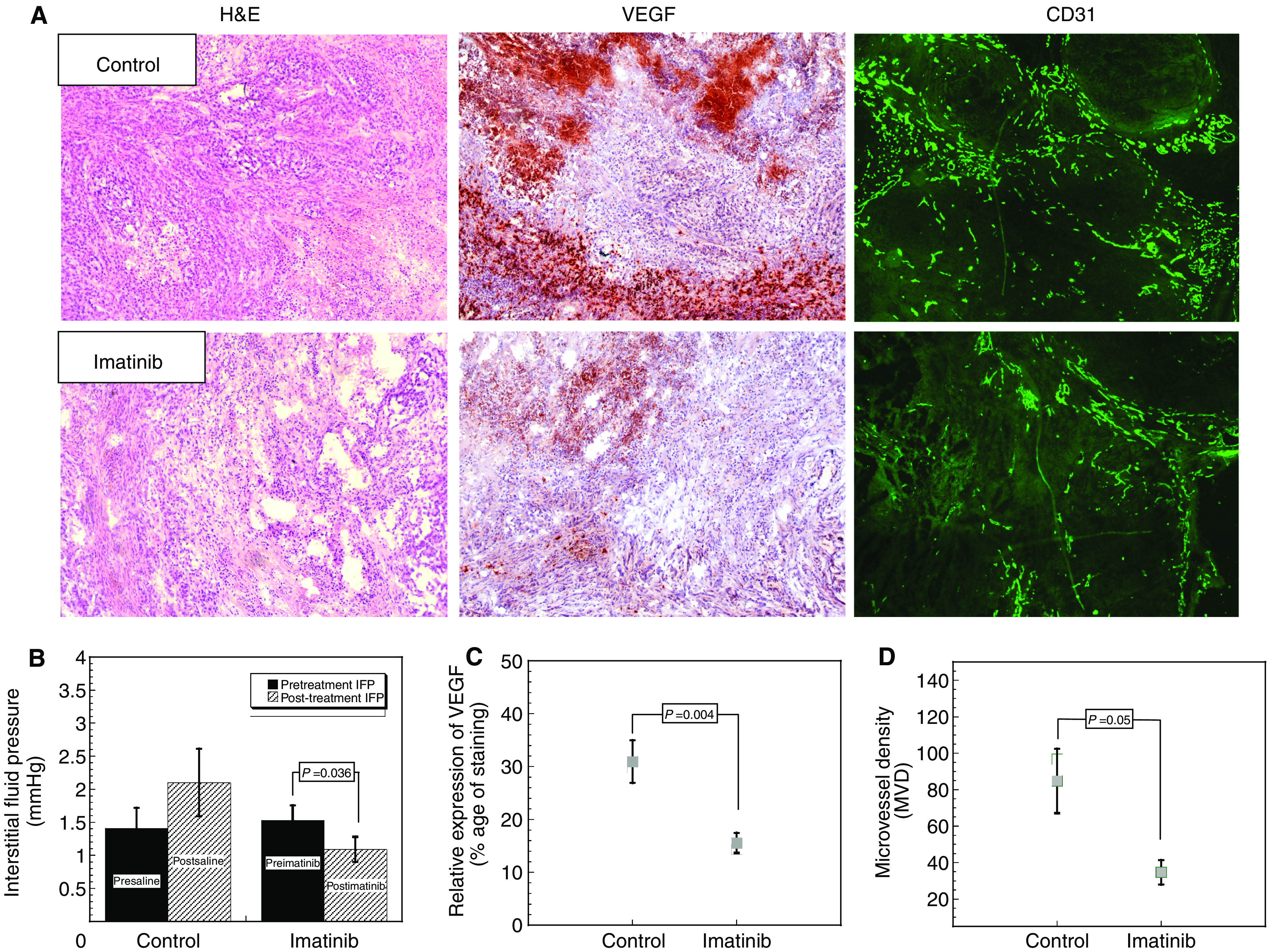
(**A**) Cross-section of NSCLC xenografts, haematoxylin and eosin stain. After treatment with Imatinib tumour, stroma appears less compacted and has loosened interstitial space compared to control; VEGF expression in control and in Imatinib-treated NSCLC xenografts; and vascular density measured by CD31 staining. (**B**) Tumour interstitial fluid pressure after 4 days of Imatinib therapy. (**C**) VEGF expression in Imatinib-treated groups (mean±s.e.m.). (**D**) Microvessel density after treatment with Imatinib (mean±s.e.m.).

To determine whether downregulation of p-PDGFR-*β* affects tumour angiogenesis, immunohistochemisty studies were performed to determine VEGF and CD31 expression (Figure 2A). VEGF was located primarily intracellularly, with less intense staining in tumour stroma. After treatment with Imatinib, VEGF expression was reduced from 31 to 15% positive area (*P*=0.004) (Figure 2C). Mean vessel density was reduced in Imatinib-treated animals. Mean vessel density averaged 84±17.7 and 44.1±6.7 (mean±s.e.m.) vessels per low-power field in control and Imatinib-treated animals, respectively. This difference was statistically significant (*P*=0.05) (Figure 2D).

A change in tumour stromal density was also observed. Figure 2A illustrates tumour stroma observed in the Imatinib-treated and control groups, respectively (haematoxylin and eosin stain). Stroma appears less dense in the Imatinib-treated *vs* the control group. Interstitial fluid pressure measurements were taken before and after 4 days of treatment. In controls, IFP averaged 1.4 and 2.1 mmHg before and after sham treatment, respectively. This difference was not significant (*P*=0.27). The Imatinib group exhibited similar pre-treatment values (1.53±0.23 mmHg). However, after 4 days of Imatinib treatment, IFP was reduced to 1.09±0.2 mmHg. This difference was statistically significant (*P*=0.036) (Figure 2B).

Imatinib-treated animals showed decreased levels of tumour hypoxia, as assessed by EF5 immunohistochemistry (Figure 1A). The percentage areas positive for EF5 were 13±2.25 and 5±1.37% (mean±s.e.m.) for the control and Imatinib-treated groups, respectively (*P*=0.008) (Figure 1C).

**Figure 3:**
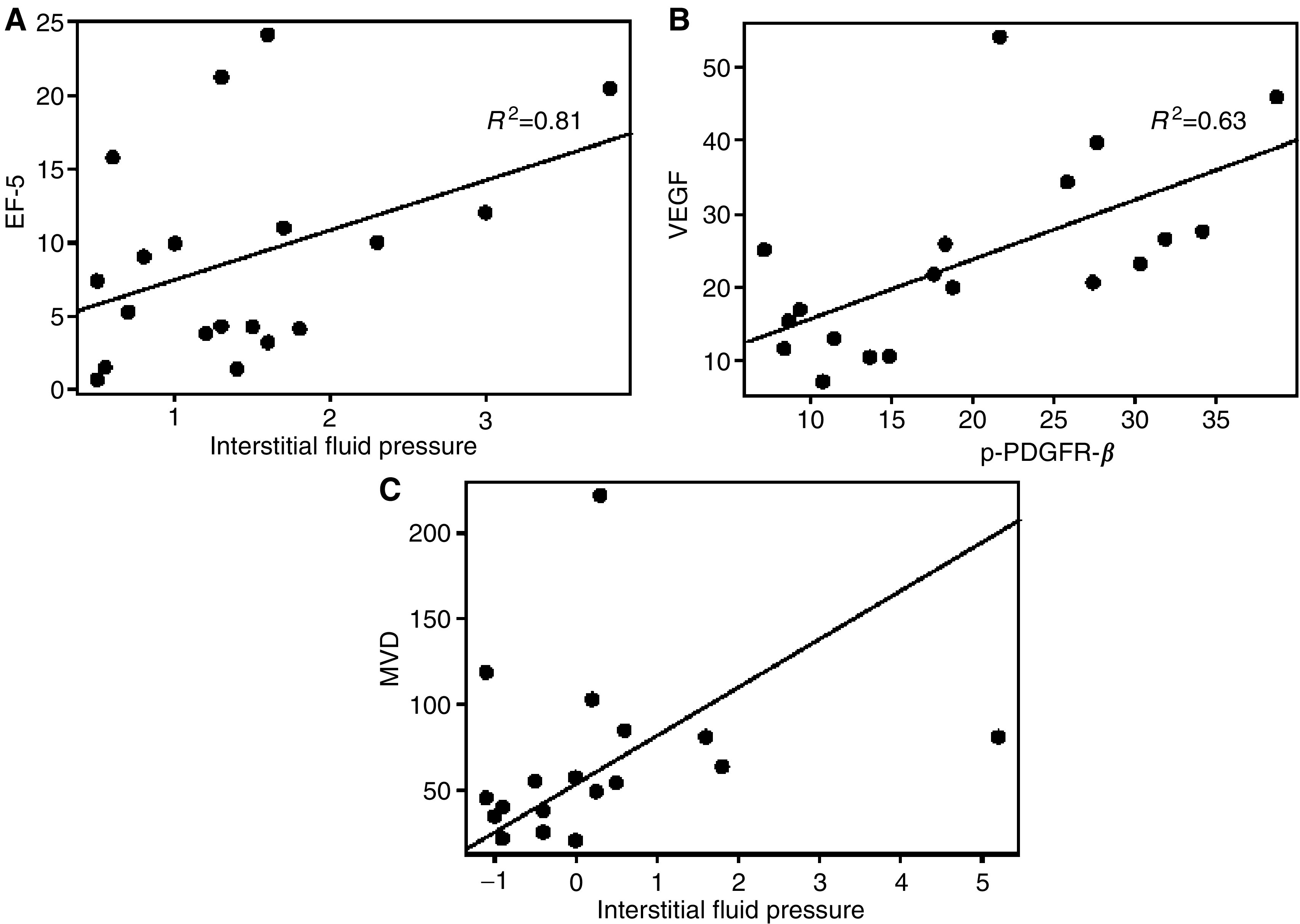
(**A**) IFP was positively correlated with EF5 (*R*^2^=0.63, *P*=0.008). (**B**) Higher levels of p-PDGFR-*β* were associated with higher levels of VEGF (*R*^2^=0.63, *P*=0.004). (**C**) There was a positive correlation between increases in IFP and levels of MVD (*P*=0.01).

Relationships between IFP, p-PDGFR-*β*, EF5, VEGF and MVD were examined after 4 days of Imatinib treatment. Interstitial fluid pressure was positively correlated with EF5 staining (*R*^2^=0.81, *P*=0.008; Figure 3A). In addition, a positive correlation was found between p-PDGFR-*β* and VEGF (*R*^2^=0.63, *P*=0.004; Figure 3B).

Further, changes in IFP were also tested for correlation with immunohistochemical parameters. A statistically significant relationship was observed between the change in IFP and MVD (*P*=0.01) (Figure 3C). This finding suggests that human NSCLC xenografts with higher MVD are more likely to exhibit a decrease in IFP following treatment with Imatinib.

## DISCUSSION

To our knowledge, this is the first study to demonstrate that the use of Imatinib in NSCLC xenografts results in decreased p-PDGFR-*β* and VEGF expression. These changes in turn affected angiogenesis (i.e. MVD), IFP and oxygenation. The increase in tumour oxygenation following Imatinib treatment was most likely due to a combination of these effects.

Aberrant tumour angiogenesis results in tortuous, leaky and nonfunctional tumour vasculature. The aberrancies in vascular structure and function play a crucial role in tumour growth and metastasis. For instance, therapeutic drug delivery is impaired thereby reducing treatment effectiveness and patient prognosis (Folkman, 1995).

Vascular smooth muscle cells and pericytes are essential for early growth of neovessels (Carmeliet, 2000). Phosphorylated PDGFR-*β* plays a crucial role in smooth muscle cell and pericyte recruitment. In normal angiogenic processes, interaction of these cells with ECs consequently results in stabilisation of the capillary wall (Hellstrom *et al*, 1999). VEGF, an EC-specific angiogenic cytokine, has been shown to induce EC proliferation and migration, increase vascular permeability, stimulate disassociation of pericytes from vascular endothelium (Morikawa *et al*, 2002) and act as a key survival factor for EC (Gerber *et al*, 1998). It has been shown that simultaneous inhibition of multiple key regulatory factors of angiogenesis (p-PDGFR-*β*, its ligand PDGF and VEGF) will decrease the percentage of tumour vessels with pericyte coverage, induce EC apoptosis and tumour regression (Shaheen *et al*, 2001). Interestingly, more mature vessels are largely resistant to p-PDGFR-*β* and VEGF inhibition (Bergers *et al*, 2003).

Data from this study shows that Imatinib reduces tumour angiogenesis, presumably through simultaneous p-PDGFR-*β* inhibition and VEGF downregulation. Whether or not this effect occurs preferentially on more immature blood vessels cannot be determined from this work because CD31, the EC marker used in this study, stains both mature and immature ECs.

Increased IFP within a tumour is a result of the tumour's highly permeable microvascular network and the absence of a functioning lymphatic system. Solid stress, generated by tumour cells growing in a confined space, also contributes to elevated IFP (Netti *et al*, 1996; Griffon-Etienne *et al*, 1999). At steady state, the pressure difference between the intravascular and interstitial spaces is near zero (Boucher and Jain, 1992). The loss of a pressure gradient across the vessel wall hinders capillary-to-interstitium drug transport (Netti *et al*, 1999). Increased IFP relates to a greatly diminished pressure differential between tissue and capillaries than exists under normal physiological conditions. The results from this study strongly support existing preclinical data demonstrating that IFP can be lowered with p-PDGFR-*β* inhibition using Imatinib (Pietras *et al*, 2001). Our data showed that in Imatinib-treated NSCLC tumours, the abundance of tumour stroma was significantly lowered, which might, as a result, impact intratumoral pressure (Figure 2A).

Tumour hypoxia is an independent predictive factor for distant metastasis and poor treatment outcome. Hypoxia can lead to ionising radiation and chemotherapy resistance by depriving tumour cells of the oxygen essential for the cytotoxic activities of these agents (Airley *et al*, 2005; Greijer *et al*, 2005). Furthermore, tumour hypoxia is associated with poor overall and disease-free survival, greater disease recurrence and less locoregional control in head and neck cancer, cervical carcinoma and soft-tissue sarcomas (Brizel *et al*, 1999; Birner *et al*, 2000; Bachtiary *et al*, 2003; Harrison and Blackwell, 2004). In addition, a statistically significant relationship between IFP and tumour oxygenation has been demonstrated in breast cancer patients (Taghian *et al*, 2005). In an animal model of metastatic prostate cancer, animals treated with Imatinib plus paclitaxel had significantly smaller tumours, and fewer bone and lymph node metastasis than mice treated with placebo or paclitaxel alone (Uehara *et al*, 2003). These mice also expressed less activated PDGFR in tumour and tumour-associated ECs, had less tumour cell proliferation and had significantly more apoptotic cells than control mice (Uehara *et al*, 2003). Statistical analysis of our data indicated a significant, linear relationship between tumour IFP and hypoxia, as measured by EF5 hypoxia marker.

**Figure 4:**
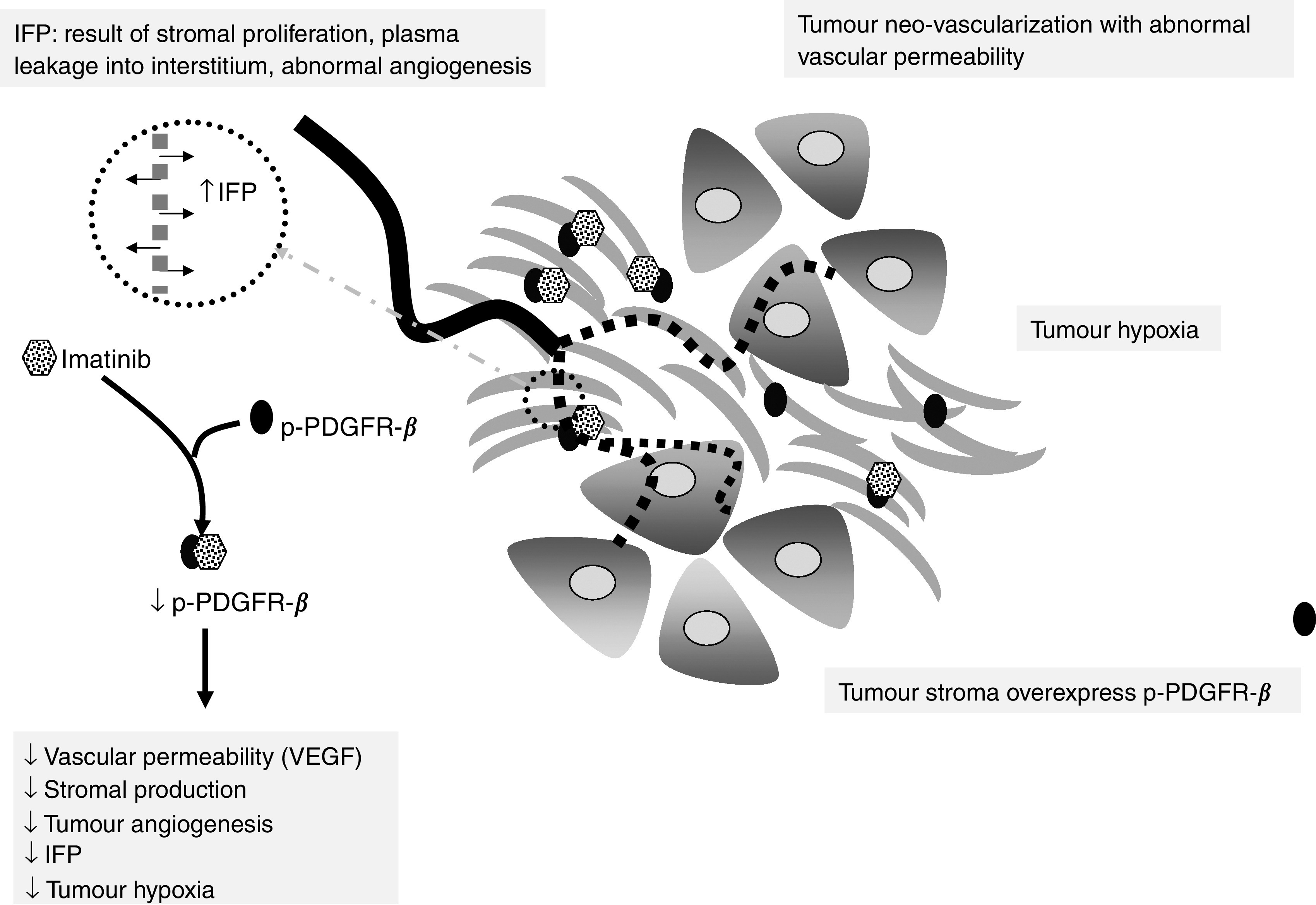
Schematic diagram illustrating the proposed mechanism of action by Imatinib. Phosphorylated platelet-derived growth factor receptor-*β* inhibition and VEGF downregulation decreases stromal production, vascular permeability, tumour hypoxia and IFP.

We argue that drug delivery could be improved by p-PDGFR-*β* inhibition and decrease of IFP. A clinical trial investigating the role of Imatinib as an adjunct to chemotherapy in patients with metastatic, PDGFR-*β*-positive, NSCLC is ongoing. The working hypothesis of the study is that blockade of PDGFR-*β* will change IFP and subsequently effect tumour perfusion/permeability, drug delivery and treatment response. Further, by improving tumour oxygenation, we hope to enhance the cytotoxic and radiation therapeutic effect in cancer patients, improve patients' treatment response and increase overall survival. Further preclinical studies are in place to evaluate a potentially pertinent clinical role for a p-PDGFR-*β* inhibitor as a radiation sensitiser (Figure 4).
